# Diagnostic Performance of Deep Learning Applications in Hepatocellular Carcinoma Detection Using Computed Tomography Imaging

**DOI:** 10.5152/tjg.2024.24538

**Published:** 2024-12-30

**Authors:** Enes Şahin, Ozan Can Tatar, Mehmet Eşref Ulutaş, Sertaç Ata Güler, Turgay Şimşek, Nihat Zafer Utkan, Nuh Zafer Cantürk

**Affiliations:** 1 Department of General Surgery, Kocaeli University Faculty of Medicine, Kocaeli, Türkiye; 2 Department of Information Systems Engineering, Kocaeli University Faculty of Technology, Kocaeli, Türkiye; 3 University of Health Science, Gaziantep City Hospital, General Surgery, Gaziantep, Türkiye

**Keywords:** Artificial intelligence, computed tomography, deep learning, hepatocellular carcinoma, liver

## Abstract

**Background/Aims:**

Hepatocellular carcinoma (HCC) is a prevalent cancer that significantly contributes to mortality globally, primarily due to its late diagnosis. Early detection is crucial yet challenging. This study leverages the potential of deep learning (DL) technologies, employing the You Only Look Once (YOLO) architecture, to enhance the detection of HCC in computed tomography (CT) images, aiming to improve early diagnosis and thereby patient outcomes.

**Materials and methods:**

We used a dataset of 1290 CT images from 122 patients, segmented according to a standard 70:20:10 split for training, validation, and testing phases. The YOLO-based DL model was trained on these images, with subsequent phases for validation and testing to assess the model’s diagnostic capabilities comprehensively.

**Results:**

The model exhibited exceptional diagnostic accuracy, with a precision of 0.97216, recall of 0.919, and an overall accuracy of 95.35%, significantly surpassing traditional diagnostic approaches. It achieved a specificity of 95.83% and a sensitivity of 94.74%, evidencing its effectiveness in clinical settings and its potential to reduce the rate of missed diagnoses and unnecessary interventions.

**Conclusion:**

The implementation of the YOLO architecture for detecting HCC in CT scans has shown substantial promise, indicating that DL models could soon become a standard tool in oncological diagnostics. As artificial intelligence technology continues to evolve, its integration into healthcare systems is expected to advance the accuracy and efficiency of diagnostics in oncology, enhancing early detection and treatment strategies and potentially improving patient survival rates.

**Main Points**

- Hepatocellular carcinoma (HCC) is the most common type of primary liver cancer, predominantly arising in patients with underlying chronic liver disease and cirrhosis.
- Computed tomography is one of the most commonly used imaging modalities in the diagnosis of HCC.
- Due to the variable tumor structure of HCC, diagnosis is sometimes difficult, additional diagnostic methods are needed, and there are delays in diagnosis and treatment.
- By integrating state-of-the-art artificial intelligence techniques with a rigorous, histopathology-validated approach, this study aims to set a new standard in HCC diagnostics, potentially transforming early detection and improving patient outcomes significantly.
- Our study illustrates the significant promise of DL models in the diagnosis of HCC, setting a foundation for future research and implementation in clinical practice.

## Introduction

Hepatocellular carcinoma (HCC) is the most common type of primary liver cancer, predominantly arising in patients with underlying chronic liver disease and cirrhosis.^1^^-^^3^ It represents a significant global health burden, ranking as the sixth most common cancer and the fourth leading cause of cancer-related deaths worldwide. The high mortality rate associated with HCC is largely attributable to late-stage diagnosis and the asymptomatic nature of the disease in its early stages.^4^^-^^8^

Early detection of HCC is vital. When diagnosed at an early stage, HCC can often be treated effectively with curative options such as surgical resection, liver transplantation, and ablative therapies.^9^^-^^12^ However, the window for such treatments is narrow, and early-stage tumors often go undetected due to their asymptomatic nature and the limitations of current diagnostic modalities. Computed tomography (CT) scans are widely used for the diagnosis and surveillance of HCC. They are crucial for detecting, staging, and guiding the treatment of liver lesions. However, CT scans have limitations, especially in identifying smaller tumors and early stage HCC.^13^^-16^ These limitations are more pronounced when CT scans are performed for indications other than HCC surveillance, as small liver tumors can be easily missed. Moreover, diagnosing HCC based solely on CT images can be complex due to the variable appearance of tumors, often leading to potential additional diagnostic workup and resulting in delays in diagnosis and definitive treatment.^13^

Deep learning (DL), a subset of artificial intelligence (AI), has shown significant promise in medical imaging.^17^ ^18^ By utilizing advanced algorithms and large datasets, DL models can identify patterns and anomalies in medical images with high accuracy. For instance, a study developed a hierarchical fusion strategy of DL networks to detect and segment HCC from dynamic CT images, achieving a global dice score of 82.8%.^19^ Another research effort reviewed the diagnostic performance of DL methods based on medical images for HCC, reporting a pooled sensitivity of 89% and specificity of 90%.^20^ Among various DL models, the You Only Look Once (YOLO) object detection algorithm stands out for its efficiency and accuracy in real-time image analysis. Example applications of YOLO show great potential in the domain.^21^^-^^23^ You Only Look Once’s ability to rapidly identify and localize objects within an image makes it particularly suited for medical imaging tasks like detecting liver tumors in CT scans. For example, a study proposed a flexible 3-dimensional hetero-phase CT HCC detection algorithm based on YOLO, demonstrating its adaptability across various imaging phases.^24^

This study aims to develop a DL-based diagnostic tool utilizing YOLO for the detection of HCC in CT scans. Our hypothesis is that this model will show excellent precision in identifying even smaller liver tumors, facilitating early and accurate HCC detection. Recognizing that histopathology is the gold standard for HCC diagnosis, our study employs stringent selection criteria. We include patients whose diagnoses have been confirmed through postoperative histopathological examination. This approach ensures that the training and validation of our DL model are grounded in the most reliable diagnostic benchmark, enhancing the model’s accuracy and clinical applicability.

By integrating state-of-the-art AI techniques with a rigorous, histopathology-validated approach, this study aims to set a new standard in HCC diagnostics, potentially transforming early detection and improving patient outcomes significantly.

## Materials and Methods

This retrospective study included 250 patients who underwent liver surgery at Department of General Surgery of Kocaeli University Faculty of Medicine from 2009 to 2023. Kocaeli University Faculty of Medicine Non-Interventional Clinical Research Ethics Committee approval was obtained (December 19, 2023, 2023/401). Written informed consent was given by the patients who agreed to participate in the study. Patients were eligible for inclusion if they were over 18 years of age and had undergone liver tumor resection, metastasectomy, or ablation surgery, with histopathological specimens collected during the procedures. Exclusion criteria included incomplete preoperative evaluations, external imaging and laboratory tests, or follow-ups conducted outside our center. Ultimately, 122 patients met the inclusion criteria, comprising 34 with HCC and 88 with other liver lesions.

### Data Preparation and Annotation

The dataset consisted of 1290 CT images divided into training (70%), validation (20%), and testing (10%) sets. Images were annotated using LabelImg, with bounding boxes marking liver lesions. Two experienced hepatobiliary surgeons performed the initial annotations, with a third surgeon independently verifying them for accuracy and consistency. All lesions were categorized as “HCC” or “other” based on histopathological confirmation.

### Model Selection and Architecture

The YOLOv8 model was selected for its optimal balance between real-time object detection efficiency and high accuracy. Its capability to rapidly identify and localize small, clinically ambiguous lesions made it particularly suitable for HCC detection. The model selection process involved an iterative evaluation of multiple architectures, including YOLOv5, YOLOv7, and YOLOv8, based on precision-recall metrics and computational efficiency benchmarks, ensuring the chosen model met the demands of both accuracy and performance.

### Data and Image Preprocessing

Data preprocessing plays a critical role in enhancing model performance. In this study, various preprocessing techniques were applied to improve the model’s robustness and generalizability. All images were resized to 512 × 512 pixels to standardize input dimensions. Random horizontal and vertical flips, along with rotations, were used to increase image variability. Additionally, brightness and contrast adjustments were applied within a specified range to simulate different imaging conditions, while random changes in hue further enhanced the model’s resilience to color variations. Collectively, these preprocessing techniques augmented the dataset, enabling the model to perform more effectively across diverse imaging scenarios.

### Training Process

The training process utilized the preprocessing techniques described above to augment the dataset and improve model generalization. The Adam optimizer was employed, optimizing a composite loss function that combined object classification, localization, and confidence loss to enhance detection accuracy. The model was trained for 100 epochs, with early stopping implemented if the validation loss plateaued for 10 consecutive epochs to prevent overfitting. A batch size of 16 was maintained throughout the training to balance computational efficiency and model performance.

### Hyperparameters

The YOLOv8 model was trained using the following default hyperparameters: a learning rate of 0.01, a batch size of 16, an Intersection over Union (IoU) threshold of 0.7 for bounding box regression, and a confidence threshold of 0.5. These hyperparameters were chosen to balance optimal detection accuracy with computational efficiency, ensuring the model’s effectiveness in identifying and localizing liver lesions.

### Evaluation Metrics

Performance was assessed using standard metrics:

Precision, recall, and F1 score: Evaluating classification and detection balance.

Mean average precision (mAP): Computed at IoU thresholds ranging from 0.50 to 0.95.

Furthermore, sensitivity, specificity, positive predictive value (PPV), and negative predictive value (NPV) were calculated using MedCalc software.^25^ These measures provided additional insights into the model’s diagnostic performance in real-world clinical settings, distinguishing its effectiveness from standard deep learning performance metrics.

## Results

From the cohort of 122 patients, we compiled a dataset of 1290 CT images. These images were associated with postoperative histopathological data, providing a robust foundation for our study. The dataset was stratified into training (70%), validation (20%), and testing (10%) sets. Over 100 training epochs, our convolutional neural network (CNN) model, based on the YOLO architecture, was rigorously trained and iteratively refined to optimize its performance in detecting various liver lesions on CT images.

The model underwent extensive training using the designated training dataset, where it learned to recognize and classify HCC lesions accurately. After the training phase, the model’s ability to generalize was evaluated on a randomized validation dataset. This phase assessed the model’s precision and recall, which are critical metrics for understanding its efficacy in lesion detection.

The model demonstrated a high precision of 0.97216, crucial for minimizing false positives and ensuring reliable lesion detection. It also achieved a recall of 0.919, effectively identifying true positives and thereby reducing the likelihood of missed lesions. The mAP at an IoU threshold of 0.50 (mAP50) was recorded at 0.94997, indicating the model’s precision in accurately localizing lesions when the predicted bounding boxes overlap by at least 50%. Additionally, the mAP from IoU threshold 0.50 to 0.95 (mAP50-95) was 0.61855, assessing the model’s robustness in lesion localization under stricter conditions.

Furthermore, the model exhibited a sensitivity of 94.74% (95% CI: 85.38%-98.90%) and a specificity of 95.83% (95% CI: 88.30%-99.13%), illustrating its capacity to accurately detect true positives and correctly identify negatives, thereby avoiding unnecessary medical interventions. The PPV and NPV were notably high at 94.74% (95% CI: 85.58%-98.20%) and 95.83% (95% CI: 88.42%-98.58%), respectively, affirming the model’s reliability in clinical predictions. The model also demonstrated excellent diagnostic differentiation with a positive likelihood ratio of 22.74 (95% CI: 7.50-68.96) and a negative likelihood ratio of 0.05 (95% CI: 0.02-0.17), enhancing diagnostic certainty. Overall, the model achieved an accuracy of 95.35% (95% CI: 90.15%-98.27%), underscoring its superior capability in correctly classifying CT images. The disease prevalence in the studied test population was estimated at 44.18%.

**Figure 1.**
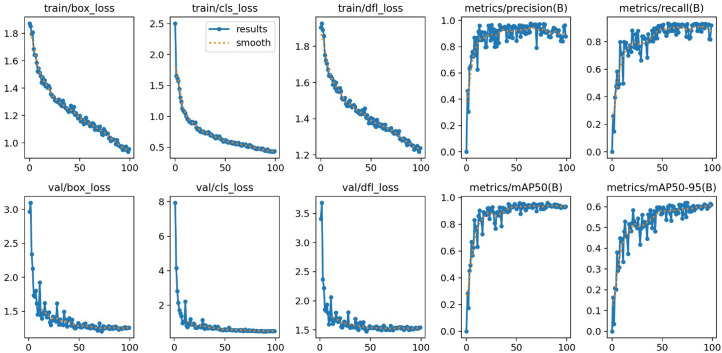
Model performance metrics.

**Figure 2.**
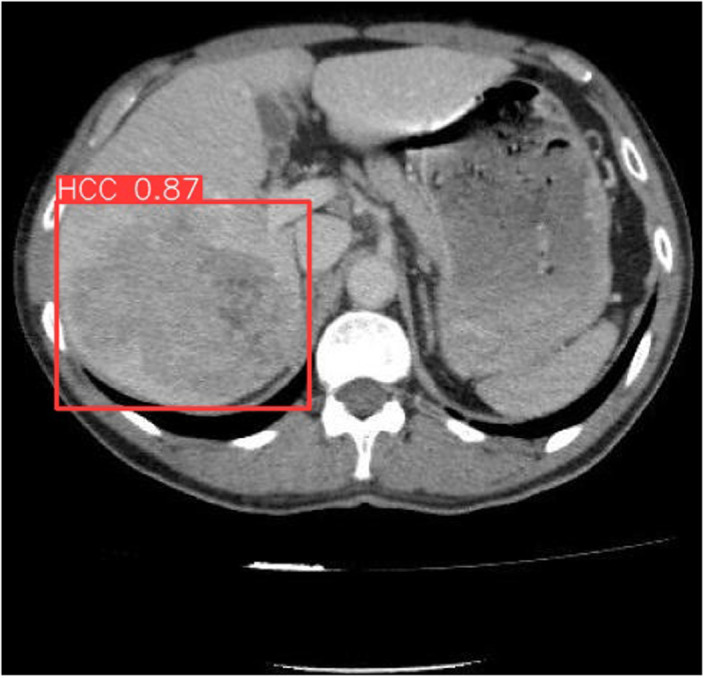
Computer tomography image of hepatocellular carcinoma.

The comprehensive results, including statistical analyses, model performance metrics, and a demonstration of the testing phase, are detailed in Table 1 and illustrated in Figures 1 and 2.

## Discussion

In this study, we employed the YOLO architecture to develop a DL model tailored for the detection of HCC in CT images. This model benefits from YOLO’s efficiency in real-time object detection, making it particularly adept at identifying and localizing liver lesions. Compared to other CNN architectures, YOLO offers substantial improvements in speed and accuracy, making it ideal for clinical environments where rapid decision-making is critical. Its application in our study has shown not only feasibility but also significant diagnostic improvements, particularly in the early detection of HCC. To our knowledge, this study represents the first application of a YOLO-based model specifically designed for detecting HCC lesions.

Our study adopts a unique protocol by exclusively utilizing cases with confirmed histopathological diagnoses, enhancing the reliability of our results. Histopathology is considered the gold standard for diagnosing conditions such as HCC. One of the primary challenges in diagnosing HCC is the misdiagnosis or failure to visualize early or difficult-to-detect lesions using traditional imaging methods. The prevalent reliance on morphological analysis from CT images, as seen in much of the existing literature, may not effectively address these challenges.^13^ In contrast, our approach leverages postoperative histopathological data as the definitive ground truth. This allows us to explore novel patterns that might be discernible in CT scans when analyzed with a DL model designed to predict surgical outcomes. Our method posits that radiological data, often intricate and laden with subtle patterns, can reveal more when examined with advanced AI technologies than what might be immediately obvious to the human eye. Furthermore, current research often relies on open-source datasets that primarily focus on radiological reports. By contrast, our study emphasizes the creation of a dataset that includes complex and challenging images, particularly those that may initially confound traditional diagnostic methods. Our dataset is notably diverse, containing primary tumors of various sizes and appearances, and spans a range of lesion-to-background contrasts (hyper-/hypo-dense), enriching our analysis and providing a robust testbed for our DL model. This approach aims to enhance the diagnostic accuracy for early or ambiguous HCC lesions, potentially leading to improved outcomes through earlier and more precise intervention.

In our study, we opted to use CT scanning as the primary diagnostic tool for detecting HCC due to its widespread availability and lower cost compared to magnetic resonance imaging (MRI), which, despite its higher diagnostic accuracy, is more expensive and less accessible.^23^ Computed tomography scans are frequently performed for various indications, which can fortuitously allow for the early detection of HCC, a potential not always possible with MRI due to its specialized use. Additionally, in patients with known risk factors for HCC development, CT scanning is often employed following the detection of suspicious features in ultrasound examinations, providing a more definitive assessment. This widespread applicability and diagnostic utility of CT scanning motivated our decision to develop a DL model based on abdominal CT imaging, aiming to enhance the early detection and diagnosis of HCC in both routine and high-risk patient screenings. In the literature, there are various studies that use either CT or MRI for detecting liver tumors, yielding similar results in terms of diagnostic effectiveness. Our choice of CT over MRI aligns with these findings, emphasizing the practicality and accessibility of CT in a broader range of clinical settings while maintaining comparably similar diagnostic accuracy in DL modalities.

Our study’s application of the YOLO architecture for detecting HCC in CT images has demonstrated satisfactory diagnostic performance. We achieved a precision of 0.97216 and a recall of 0.919, indicative of our model’s capability to minimize false positives and effectively identify true positives. The mAP at an IoU threshold of 0.50 (mAP50) was recorded at 0.94997, showcasing precise lesion localization. Additionally, our model exhibited high sensitivity (94.74%) and specificity (95.83%). The overall accuracy of 95.35% underscores the model’s superiority in correctly classifying CT images. Comparatively, Kim et al^24^ employed a DL model with hepatobiliary phase MRI, achieving a sensitivity of 87% and specificity of 93%. Despite MRI’s generally higher diagnostic efficacy, our CT-based approach demonstrated superior performance, highlighting the effectiveness of the YOLO-based model in HCC detection. In another study, Wang et al developed a deep-learning AI system analyzing liver CT imaging data.^25^ Their model achieved an accuracy of 81.0% and sensitivity of 78.4% on the internal test set, with slightly better performance on the external test set. Gao et al introduced a DL model that differentiates between malignant hepatic tumors using multi-phase CT and clinical features, achieving a sensitivity of 0.865 and specificity of 0.868 for HCC.^26^ While their study incorporates clinical features for diagnosis, our focused approach on imaging alone still offers comparable diagnostic accuracy. Lastly, Hamm et al’s research with a custom CNN for classifying hepatic lesions on MRI demonstrated a high accuracy of 92% and specificity of 98%.^27^ While their findings are significant, they focus on MRI, which is traditionally more sensitive than CT for certain diagnoses. However, the success of our CT-based model underscores the potential of DL in enhancing diagnostics even with modalities considered less sensitive in specific contexts. Overall, our findings not only align with but often surpass outcomes from some other advanced studies, emphasizing the potential of AI, particularly DL models like YOLO and some novel approaches like curating surgically proven, complex datasets. This suggests a promising direction for future research and practical applications, where such AI models could become standard tools, enhancing the accuracy and efficiency of oncological diagnostics across various imaging modalities.

Despite its strengths, our study has limitations due to its retrospective design and single-center dataset, which may affect the generalizability of the findings. Future multicentric studies could expand the dataset to enhance the model’s broader efficacy and include a more holistic approach to liver health. By aiming to detect any liver lesions with precision and correlating imaging findings with true histopathological diagnoses, such studies would not only validate the effectiveness of the model across different populations but also improve diagnostic accuracy for a variety of liver diseases.

This study highlights the potential of DL models, particularly YOLOv8, in the diagnosis of HCC. The model demonstrated high precision, sensitivity, and specificity, effectively detecting small and early-stage liver lesions often missed by conventional imaging techniques. These results underline the transformative role of AI in improving early detection, guiding timely interventions, and enhancing patient outcomes in oncology.

Despite these promising findings, there are critical challenges to address. Future research should focus on leveraging multicentric big data to train and validate DL models. Data from multiple centers, encompassing diverse imaging protocols, patient demographics, and equipment, will ensure the robustness and generalizability of AI systems. Differences among centers, such as varying imaging quality and diagnostic practices, pose significant challenges but also offer opportunities to improve model adaptability in real-world clinical environments. Moreover, the integration of multimodal data—combining imaging with clinical, laboratory, and genomic information—could enhance diagnostic accuracy and enable personalized treatment strategies. This approach aligns with the principles of precision medicine, where AI can help tailor interventions based on individual patient profiles. In addition to technical improvements, the development of explainable AI frameworks is essential. Transparency in model decision-making will foster trust among clinicians, facilitating broader adoption. Addressing ethical considerations, including data privacy and security, will also be crucial for the responsible implementation of AI in healthcare.

In conclusion, while our findings demonstrate the promise of DL in HCC diagnosis, addressing data heterogeneity, inter-center variability, and clinical integration will be key to fully realizing its potential in transforming oncology care.

## Availability of Data and Materials:

The data that support the findings of this study are available on request from the corresponding author.

## Figures and Tables

**Table 1.** Comprehensive Results of the Model

Statistic	Value	95% CI
Sensitivity	94.74%	85.38%-98.90%
Specificity	95.83%	88.30%-99.13%
Positive likelihood ratio	22.74	7.50-68.96
Negative likelihood ratio	0.05	0.02-0.17
Disease prevalence on dataset	44.18%	
Positive predictive value	94.74%	85.58%-98.20%
Negative predictive value	95.83%	88.42%-98.58%
Accuracy	95.35%	90.15%-98.27%
